# Value of Routine Magnetic Resonance Imaging for the Preoperative Assessment of Cochlear Implant Candidates

**DOI:** 10.7759/cureus.6279

**Published:** 2019-12-03

**Authors:** Farid Alzhrani, Alaa Babkour, Fida Almuhawas, Abdulrahman Sanosi

**Affiliations:** 1 Otolaryngology and Neurotology, King Abdullah Ear Specialist Center, King Saud University, Riyadh, SAU; 2 Otolaryngology, Al Noor Specialist Hospital, Makkah, SAU; 3 Otolaryngology, King Abdullah Ear Specialist Center, King Saud University, Riyadh, SAU; 4 Otolaryngology, Neurotology and Skull Base Surgery, King Saud University, Riyadh, SAU

**Keywords:** cochlear implantation, high-resolution computed tomography, magnetic resonance imaging, preoperative assessment, temporal bone

## Abstract

Background

The selection of an appropriate imaging technique for assessment before cochlear implantation is critical for precise diagnosis and management. While magnetic resonance imaging (MRI) is used for the diagnosis of several conditions, such as labyrinthitis ossificans, cochlear nerve deficiency, and neoplasms, high-resolution computed tomography (HRCT) provides excellent details of the temporal bone. However, it remains unclear whether routine MRI provides any additional benefits over HRCT.

Objectives

To assess the added value of MRI as a screening tool for temporal bone abnormalities in cochlear implant candidates through comparisons of its findings with those of HRCT.

Materials and method

It is a retrospective analysis of preoperative HRCT and MR images in a tertiary referral center. A total of 308 patients who underwent MRI and HRCT examinations before cochlear implantation between 2013 and 2015 were included. Preoperative HRCT and MR images were screened for temporal bone abnormalities by a senior neurotologist and a neuroradiologist.

Results

HRCT detected inner ear deformities in 51 of the 308 (16.6%) subjects, whereas MRI revealed abnormalities in only 18 (5.8%) of subjects. HRCT detected the same inner abnormalities in 16 of the 18 (88.9%) subjects diagnosed by MRI, whereas it showed normal results for the remaining two subjects. MRI detected cochlear nerve aplasia/hypoplasia in 13 subjects, 11 of whom had associated inner ear deformities that were detected by HRCT. The MR images of nine subjects showed cochlear fibrosis, which was confirmed by HRCT in all nine subjects.

Conclusion

In this study, MRI did not exhibit significant additional benefits over HRCT, and its routine use for the preoperative assessment of CI candidates was not justified. However, MRI is warranted for subjects at an increased risk of cochlear nerve aplasia due to an inner ear deformity or a narrow internal auditory canal. The establishment of criteria that facilitate the performance of MRI only when absolutely needed will reduce healthcare costs, prevent unnecessary exposure to the risks associated with general anesthesia, and shorten delays before cochlear implantation.

## Introduction

Cochlear implantation greatly influences the lives of individuals with deafness and their families because it allows the recipients to achieve excellent levels of communication and interaction with their surroundings [1]. At our institution, magnetic resonance (MR) imaging (MRI) and high-resolution computed tomography (HRCT) are requested for all cochlear implant (CI) candidates. While HRCT provides excellent details of the temporal bone anatomy [1-2], MRI can provide information about the contents of the internal auditory canal (IAC), specifically the cochlear nerve, and the patency of the cochlea [1-3].

However, there is some controversy surrounding the use of MRI as a routine tool for assessments before cochlear implantation. Fishman [1] advocated HRCT as the initial imaging modality of choice and suggested that MRI may be a useful additional tool in select cases. Other authors have suggested that both MRI and HRCT are essential components of preoperative assessments for CI candidates [2,4]. Meanwhile, Mackeith et al. [5] and Parry et al. [6] asserted that MRI is generally the only required imaging examination before cochlear implantation. According to these authors, MRI is less likely to miss an abnormality than is HRCT. However, they also stated that HRCT may be required in certain cases such as in those with a history of severe middle ear disease, meningitis, or dysmorphic syndromes.

MRI is superior to HRCT in terms of early and late diagnoses of labyrinthitis ossificans, the identification of cochlear nerve deficiency, and the evaluation of neoplasms or processes that affect the central auditory pathways [2,6-7]. Nevertheless, the added value of routine MRI for the preoperative assessment of CI candidates remains questionable, particularly because the only contraindications for cochlear implantation are labyrinthine/cochlear aplasia and the absence of the auditory nerve, which (along with the narrowing of the IAC by <2.5-3.0 mm) can be predicted by HRCT [1,3].

Because most of the CI candidates at our institution are children, the technical difficulties in performing a lengthy and demanding procedure, such as MRI, necessitates general anesthesia. Furthermore, the long waiting time for MRI in some centers can delay implantation and, for pediatric candidates, waste precious time during which language acquisition is optimal [8-11]. Therefore, the aim of the present study was to assess the added value of routine MRI as a screening tool for temporal bone abnormalities in CI candidates through comparisons of its findings with those of HRCT.

## Materials and methods

Ethics

This retrospective cohort study was approved by the Institutional Review Board (IRB) of the College of Medicine, King Saud University.

Cohort

We retrospectively evaluated the data for all subjects whose files were presented to the cochlear implantation committee between February 2013 and March 2015. A total of 308 (162 male and 146 female) subjects, including 268 children aged two to 18 years and 40 adults who underwent both MRI and HRCT before cochlear implantation, were enrolled.

Imaging techniques

HRCT was performed using a 64-slice scanner in the axial plane with a 0.625-mm slice thickness. MRI was performed using 3T equipment. T1-weighted and T2-weighted spin-echo images as well as heavily T2-weighted sequences, namely, fast imaging employing steady-state acquisition (FIESTA) or constructive interference in steady-state (CISS) sequences, were acquired. All MR and HRCT images and their findings were screened twice by a senior neurotologist and a neuroradiologist in the Centricity System (GE Healthcare, Illinois).

The latest classification system of Sennaroglu [3] was used for the assessment and categorization of inner ear abnormalities. The width of IAC was measured in the axial plane, 2 mm within the medial lip between the anterior and posterior walls [9].

Data analysis

All data were analyzed using Microsoft Excel 2011, version 14.0.0 (Microsoft Corporation, Washington).

## Results

**Figure 1:**
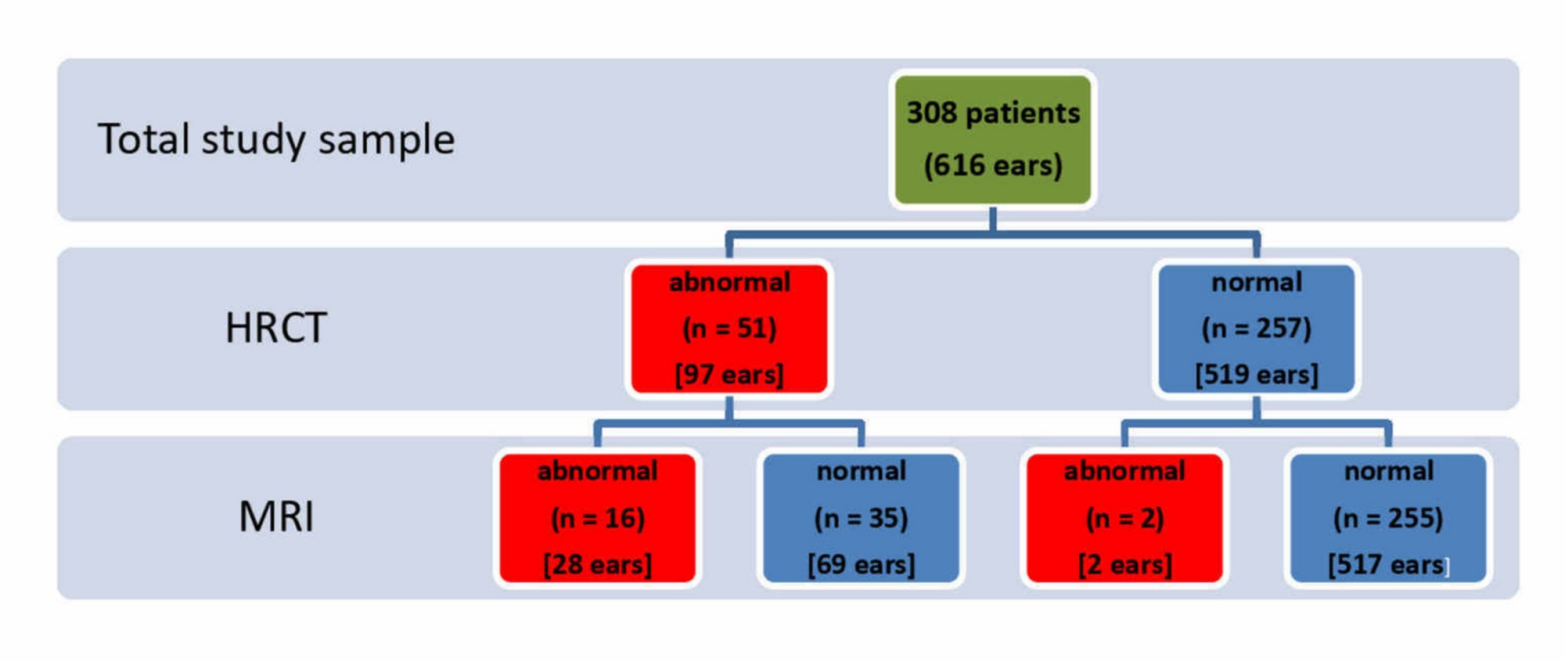
Abnormal findings in radiological examinations conducted before cochlear implantation in a cohort of pediatric and adult subjects HRCT, high-resolution computed tomography; MRI, magnetic resonance imaging

During HRCT screening, 51 of the 308 (16.6%) subjects exhibited inner ear deformities (Figure 1); these included 44 (14.3% of the total) subjects with similar bilateral deformities, five (1.6% of the total) with a unilateral deformity, and two (0.6% of the total) with a different deformity on each side. Cochlear deformities were detected in 27 of the 308 (8.8%) subjects. There were 22 (7.1% of the total), three (1.0% of the total), and two (0.6% of the total) subjects with similar bilateral deformities, a unilateral deformity, and a different deformity on each side, respectively.

MRI screening revealed abnormalities in 18 of the 308 (5.8%) subjects, including 12 (3.9% of the total) with bilateral abnormalities and six (1.9% of the total) with a unilateral abnormality. HRCT detected the same inner abnormalities in 16 of these 18 (88.9%) subjects, whereas it showed normal results for the remaining two subjects.

MRI identified 13 (4.2% of the total) subjects with cochlear nerve aplasia/hypoplasia (bilateral, n = 8; unilateral, n = 5; Table 1), 11 of whom (3.6% of the total) had associated inner ear deformities that were detected by HRCT. The remaining two (0.6% of the total) subjects showed no inner ear abnormalities on HRCT images (Table 1). The mean IAC diameter in the 13 subjects was 2.44 mm (range: 1.14-4.84 mm), and nine of them showed an absent or narrow (≤3 mm) IAC (bilateral, n = 6; unilateral, n = 3).

**Table 1:** Subjects who exhibited cochlear nerve aplasia/hypoplasia on magnetic resonance images obtained before cochlear implantation IAC, internal auditory canal; A, aplastic; IP, incomplete partition * Mean width of the internal auditory canal was ≥3 mm. ** Represents subjects with A/Hypoplastic cochlear nerve on magnetic resonance imaging with no inner ear abnormalities on high-resolution computed tomography.

Subject	Side	Cochlear nerve	Inner ear deformity (if present)	IAC width
1	Right	A/Hypoplastic	Type-I IP	2.26 mm
	Left	A/Hypoplastic	Type-I IP	2.61 mm
2	Right	A/Hypoplastic	Type-I IP	Normal^*^
	Left	A/Hypoplastic	Type-I IP	Normal^*^
3	Right	A/Hypoplastic	Cochlear Hypoplasia	1.42 mm
	Left	A/Hypoplastic	Cochlear Hypoplasia	1.20 mm
4	Right	A/Hypoplastic	Cochlear Hypoplasia	Normal^*^
	Left	Normal	Normal	Normal^*^
5	Right	A/Hypoplastic	Cochlear Aplasia	2.13 mm
	Left	A/Hypoplastic	Cochlear Aplasia	1.14 mm
6	Right	A/Hypoplastic	Michel Aplasia	Absent IAC
	Left	A/Hypoplastic	Michel Aplasia	Absent IAC
7	Right	A/Hypoplastic	Narrow IAC	2.58 mm
	Left	A/Hypoplastic	Narrow IAC	2.56 mm
8	Right	A/Hypoplastic^**^	Normal	Normal^*^
	Left	A/Hypoplastic	Narrow IAC	1.70 mm
9	Right	Normal	Normal	Normal^*^
	Left	A/Hypoplastic	Narrow IAC	1.85 mm
10	Right	A/Hypoplastic	Cochlear Aplasia	2.63 mm
	Left	Normal	Normal	Normal^*^
11	Right	A/Hypoplastic^**^	Normal	Normal^*^
	Left	Normal	Normal	Normal^*^
12	Right	A/Hypoplastic	Narrow IAC	1.90 mm
	Left	A/Hypoplastic	Narrow IAC	2.31 mm
13	Right	A/Hypoplastic^**^	Normal	Normal^*^
	Left	Normal	Normal	Normal^*^

MRI detected cochlear fibrosis in nine of the 308 (2.9%) subjects, four of whom had other inner ear anomalies. The remaining five subjects (bilateral, n =4; unilateral, n =1) showed evidence of a reduced T2 signal or loss of the T2 signal in the cochlear fluid. Notably, HRCT also detected the cochlear fibrosis in all nine cases.

## Discussion

In the present study, we found that routine MRI does not add value to HRCT for the detection of temporal bone abnormalities in CI candidates and that it may not be necessary for all patients.

At present, data regarding the necessary radiological investigations before cochlear implantation remain unclear, and it has been suggested that MRI is not routinely required and only necessary in cases with specific indications [1-2]. Other researchers have claimed that MRI is necessary and that some CI candidates with a normal inner ear morphology evidently have cochlear nerve aplasia [5,12-13].

The proponents of MRI suggest that it should be used as the initial imaging technique of choice because it is less likely to overlook an abnormality than is HRCT [5]. MRI can detect cochlear nerve aplasia/hypoplasia, which is an absolute contraindication for implantation and cannot be detected by HRCT. Moreover, it can evaluate neoplasms or processes affecting the central auditory pathways [2,5,7,13]. In addition, HRCT is less sensitive than MRI in the detection of labyrinthitis ossificans, particularly during the early phase of fibrosis secondary to meningitis [1-2,5].

The proponents of HRCT argue that it can directly predict labyrinthine/cochlear aplasia and indirectly predict the absence of the cochlear nerve from the presence of a narrow IAC (<2.5-3.0 mm) [1,3]. In one study, the sensitivity and specificity of HRCT for the identification of cochlear obstruction on scans with a slice thickness of 1.5 mm were 100% and 86%, respectively [1].

Most CI candidates in the present study were children; therefore, HRCT was performed with a radiation dose of 120 kV/250 mAs. MRI for pediatric patients is a difficult and lengthy process and necessitates general anesthesia. Furthermore, the typically long waiting time for MRI in some centers can delay the implantation process, thereby wasting precious time that could be used for language acquisition and speech development [8-10]. Among the multiple factors that can affect the final outcome of implantation, the age at which implantation occurs in recipients with prelingual deafness is crucial: a delay of just a few months can make a noticeable difference [8-10,12]. This emphasizes the importance of early implantation. In the present study, MRI was essentially of no value for 306 of the 308 subjects. If these subjects had received timely implantation instead of waiting for MRI, which has an average waiting time of approximately six months at our institution, they would have most likely exhibited better and more rapid language acquisition. Furthermore, all the pediatric patients could have avoided the risks associated with general anesthesia during MRI. It is primarily for these reasons that we agree with Fishman [1], who argued that HRCT should be routinely used before implantation and that MRI should only be used if a narrow IAC or any other inner ear abnormality is detected. MRI may also be warranted for the identification of labyrinthine ossificans in CI candidates with a history of meningitis, particularly if the infection was recent.

One study reported cochlear nerve aplasia or hypoplasia in 6.0%-16.1% patients with sensorineural hearing loss (SNHL) [14]. In the present study, it was detected in 4.2% of the included subjects. Only two subjects with an aplastic/hypoplastic cochlear nerve detected by MRI showed no inner ear abnormalities on HRCT. Nelson and Hinojosa [13] dissected the temporal bone and reported that the cochlear nerve could be absent despite the presence of normal inner ear structures, although they did not suggest a prevalence for this condition. Their study was based on the temporal bones of two patients with known cochlear nerve aplasia [13].

Bamiou et al. reported a mean IAC diameter of ≤3 mm for ears with cochlear nerve aplasia/hypoplasia [15], whereas the present study found a mean IAC diameter of 2.44 mm (range 1.14-4.84 mm). Accordingly, we recommend that MRI should be performed for cases where the IAC diameter is ≤ 2.44 mm.

We also observed that both MRI and HRCT detected cochlear fibrosis in nine subjects, probably because of the late presentation of these patients for the investigation of SNHL.

This study was limited by the small sample size. Nevertheless, the findings provide information that can help in limiting healthcare costs by aiding in the selection of an appropriate imaging modality that best reflects the pathophysiology of the condition. However, further multicenter studies with larger sample sizes are necessary for clarifying our findings and establishing criteria that will facilitate the performance of MRI only, when absolutely required.

## Conclusions

In conclusion, we found that MRI provided limited additional benefits over those of HRCT for the detection of temporal bone abnormalities in CI candidates; therefore, its routine use for the preoperative assessment of these candidates may not be justified. However, it can be used to confirm the presence of the cochlear nerve in patients with inner ear deformities and narrow IACs. Furthermore, it can be used to confirm suspected cochlear fibrosis. These measures will not only prevent the unnecessary use of MRI and the adverse events associated with general anesthesia but also reduce healthcare costs. Moreover, they will reduce the threshold at which patients with congenital SNHL undergo implantation, thus resulting in better and more rapid language acquisition.
